# Application of postmortem computed tomography angiography to settle a medical dispute after aortic dissection surgery: a forensic case report

**DOI:** 10.1186/s13019-023-02353-8

**Published:** 2023-08-18

**Authors:** Wang Zhuoqun, Tian Zhiling, Wan Lei, Dong Hewen, Zou Donghua, Zhang Jianhua, Liu Ningguo

**Affiliations:** 1 Shanghai Key Laboratory of Forensic Medicine, Shanghai Forensic Service Platform, Academy of Forensic Science, Ministry of Justice, Shanghai, People’s Republic of China; 2 School of Basic Medical Sciences, Shanghai University of Medicine & Health Sciences, Shanghai, China

**Keywords:** Postmortem computed tomography angiography, Cardiac surgery, Aortic dissection, Medical dispute, Forensic pathology

## Abstract

**Background:**

In the present case, we applied postmortem computed tomography angiography (PMCTA) in a medical dispute involving sudden death after cardiovascular surgery.

**Case presentation:**

A 39-year-old man underwent aortic arch replacement combined with stented elephant trunk implantation surgery under extracorporeal circulation. All vital signs were stable and he was arranged for discharge seven days after surgery. Several days later, the patient was sent back to the hospital for chest pain and poor appetite. Unfortunately, his condition worsened and he ultimately died. PMCT scanning detect pericardial effusion. Family members suspected that the surgical sutures were not dense enough, causing the patient’s postoperative bleeding and resulting in cardiac tamponade and death. PMCTA was performed before autopsy, which showed pericardial effusion. However, postmortem angiography with simulated blood pressure showed no leakage of contrast agent, which guided the subsequent autopsy and histological examinations.

**Conclusions:**

While many previous postmortem imaging case reports have shown positive results that provided evidence of medical malpractice, the current case excludes the possibility of physician negligence and reasonably settles the medical dispute from another perspective. In short, the PMCTA approach we describe here was an effective tool that can be applied to certain medical-related forensic cases.

## Background

Medical malpractice is relatively common in cardiovascular surgeries [1–3], such as aortic dissection surgery [4], and is disproportionately associated with its incidence: approximately 7.7 cases yearly per 100,000 operations [5]. Abnormal surgical operations and irregular operation of surgeons are the most direct causes of medical disputes, which often lead to doctor-patient conflicts before entering the judicial evaluation process. Among these cases, there are many involving postoperative bleeding caused by untight surgical suture [6]. A key goal of forensic pathology examinations in these instances is to determine if a surgical factor contributed to the subject’s death, potentially clarifying if any negligence occurred during the operation, especially the surgical suture process. There are high risks and technical challenges associated with cardiovascular surgery [7], as well as trauma, local tissue adhesions, and other factors that can undoubtedly increase the difficulty of the autopsy. Moreover, even if local active hemorrhage is found during the autopsy, it may be unclear or controversial in many cases whether the hemorrhage was caused by surgical negligence.

In recent years, the application of postmortem imaging techniques, such as postmortem computed tomography (PMCT) and PMCT angiography (PMCTA), is becoming increasingly common in the forensic science field [8–13]. It has been reported that angiographic techniques could be auxiliary in solving medical disputes that involve cardiovascular surgery [14, 15]. Here, we present a medical dispute case of sudden death after aortic dissection surgery using the PMCTA method.

### Case presentation

A 39-year-old man with aortic dissection was hospitalized for chest and back pain. Relevant preoperative examinations were performed and the patient underwent aortic arch replacement combined with stented elephant trunk implantation surgery under extracorporeal circulation. After exposure of the pericardial cavity, 50 mL of bloody pericardial effusion was detected. Dissection scale involving the ascending aorta and the brachiocephalic vessels of the aortic arch, diseased ascending aorta, and arch were then resected. Finally, four branch artificial blood vessels were installed. The whole operation procedure went well. The patient’s vital signs were stable and he was discharged from the hospital on the 7th day after operation. At home, he received continuous anticoagulant, diuretic, and antihypertensive therapy. On day 17 after surgery, the patient was sent back to hospital for chest pain, chest congestion, and poor appetite. His condition continuously worsened, and he died on day 18 after surgery.

Family members of the patient questioned his death, which led to disputes between them and his physician. When the family was preparing to sue the hospital, lawyers and clinicians were engaged to analyze the medical records. The clinical medical experts engaged by the family analyzed that the cause of death may be cardiac tamponade. They suspected that the sutures in the operation were not dense enough, which resulted in his postoperative bleeding and ultimately cardiac tamponade and death. This would explain why the patient had chest pain symptoms after surgery. His sudden death after a few days of chest tightness indicates that the bleeding caused compression of the heart, referred to as cardiac tamponade. The physician argued that there was nothing wrong with the surgical sutures because no bleeding was observed after the operation, even after repeated inspections. Because postoperative bleeding often emerges quickly and will not last for a few days, the patient’s death was likely from its own cause.

### PMCTA examination

External forensic examination was performed first, followed by PMCTA via the carotid artery. The end of the customized angiographic device [16] was inserted into the carotid artery towards the heart and fixed in place, while the other end facing the head was ligated to ensure that contrast fluid would flow into the aorta rather than the intracranial vessels. Contrast medium (meglumine diatrizoate and 0.9% normal saline at a 10:1 ratio) was injected using a peristaltic pump (Masterflex® L/S® Series Peristaltic Pumps, USA). The pressure was gradually increased to simulate normal blood pressure in vivo. The filling of the contrast medium in the aorta and cardiac cavity was dynamically monitored, and local contrast medium leakage should be watched closely. The target was scanned using a 40-slice multislice SOMATOM Definition AS CT system (Siemens Healthcare GmbH, Munich, Germany). Raw data were acquired using the following settings: voltage, 120 kV; current, 240 mA; and collimation, 6.0 × 1.0 mm. Image reconstruction was achieved at a 5.0 mm and 0.625 mm slice thickness, each with an increment of one-half the slice thickness. Image review and 3-dimensional reconstructions were performed using a Syngo Imaging XS CT workstation (Siemens Healthcare GmbH).

## Results

### Radiological findings

**Fig. 1:**
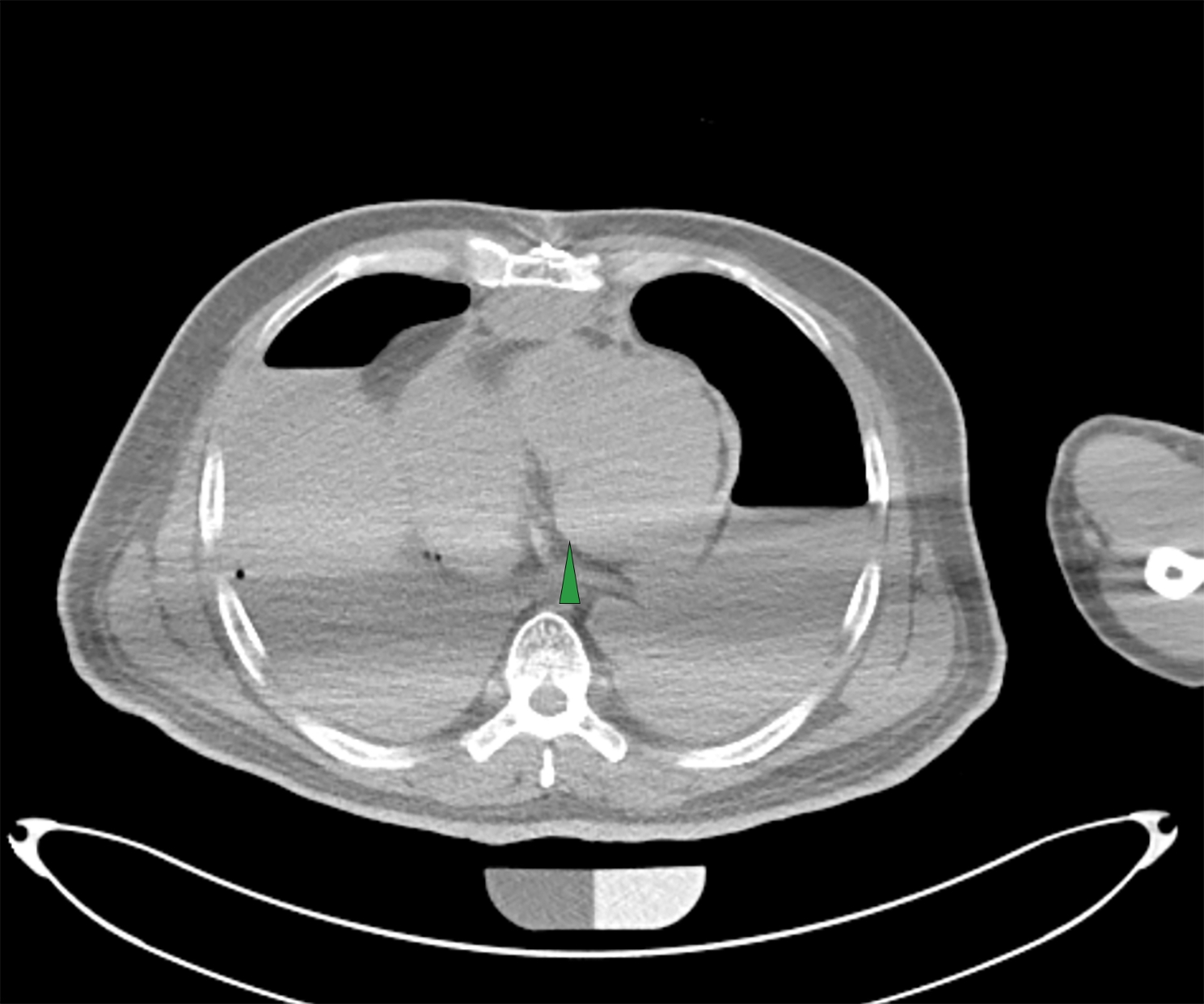
Radiological findings. Effusion was found in the pericardial cavity (arrow)

**Fig. 2:**
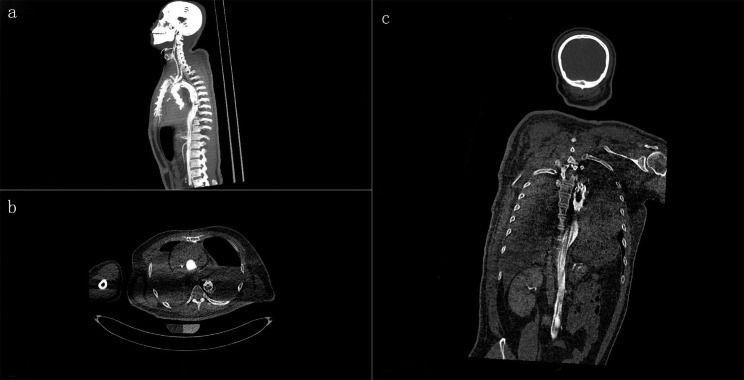
Radiological findings. The postmortem computed tomography angiography (PMCTA) results show no flow of the contrast agent from the blood vessel in the (**a**) sagittal, (**b**) axial, or (**c**) coronary aspect

**Fig. 3:**
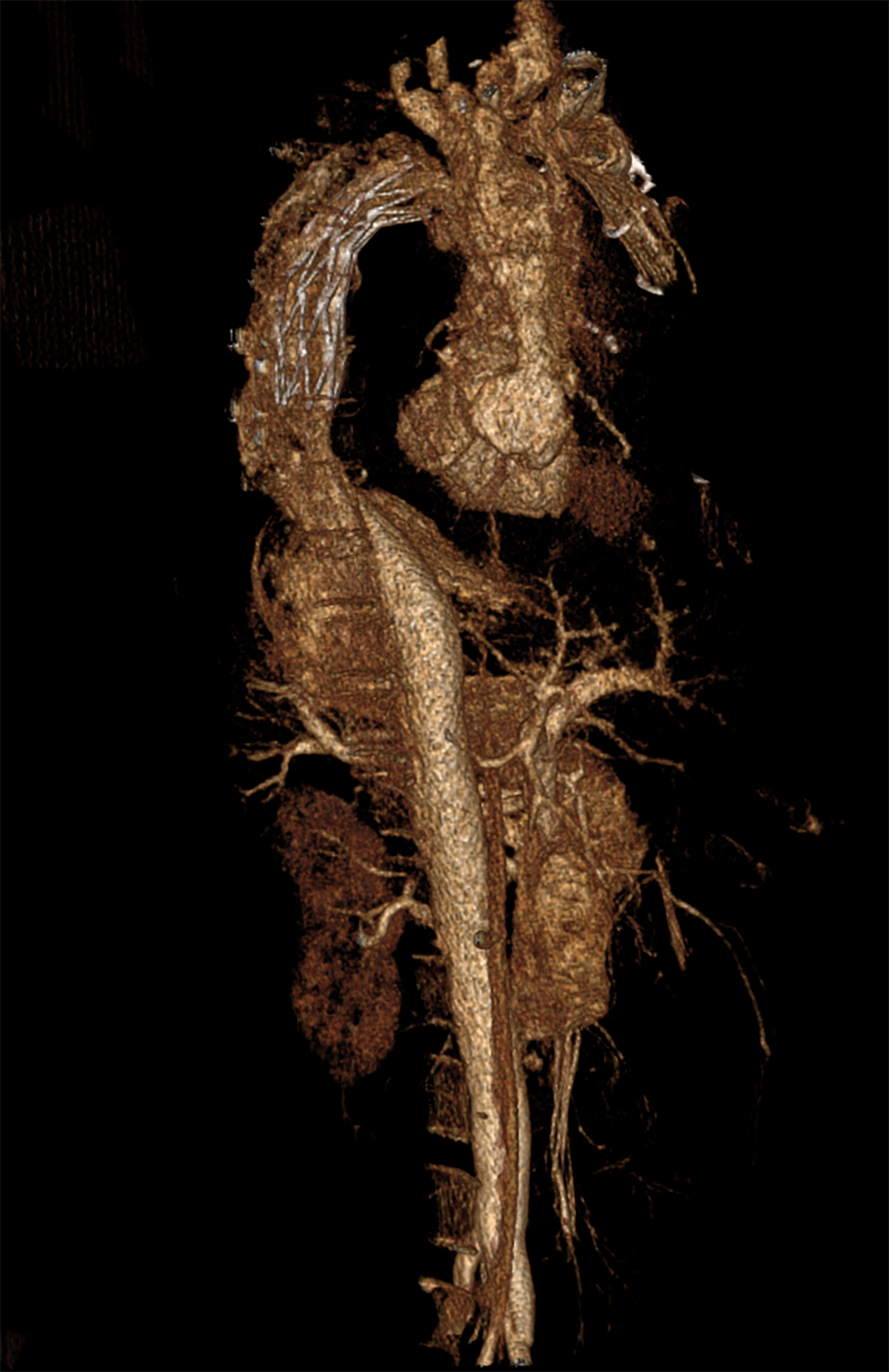
Radiological findings. Three-dimensional reconstruction of the postmortem computed tomography angiography (PMCTA) findings using a VRT. The image shows that the tiny blood vessels of the organs and tissues were filled with contrast medium and no contrast agent flowed from the blood vessel

CT scanning showed no cerebral hemorrhage or any trauma. No fatal hemorrhage from other sites was detected. Effusion was found in the pericardial cavity (Fig. 1), but because of the particularity of the image, it was impossible to judge whether the effusion was hemostatic or not. Contrast enhanced CT scanning showed that the contrast medium was filled in the aorta and heart, but there was no obvious local leakage (Fig. 2). The contrast medium was continuously injected until the pressure reached in vivo blood pressure levels. The results show that the tiny blood vessels of organs and tissues were filled with contrast medium, and a small amount of contrast medium was diffusely leaked. This is from the increased vascular permeability after death. However, there was no obvious crevasse in the local area (Fig. 3).

### Autopsy and histological findings

**Fig. 4:**
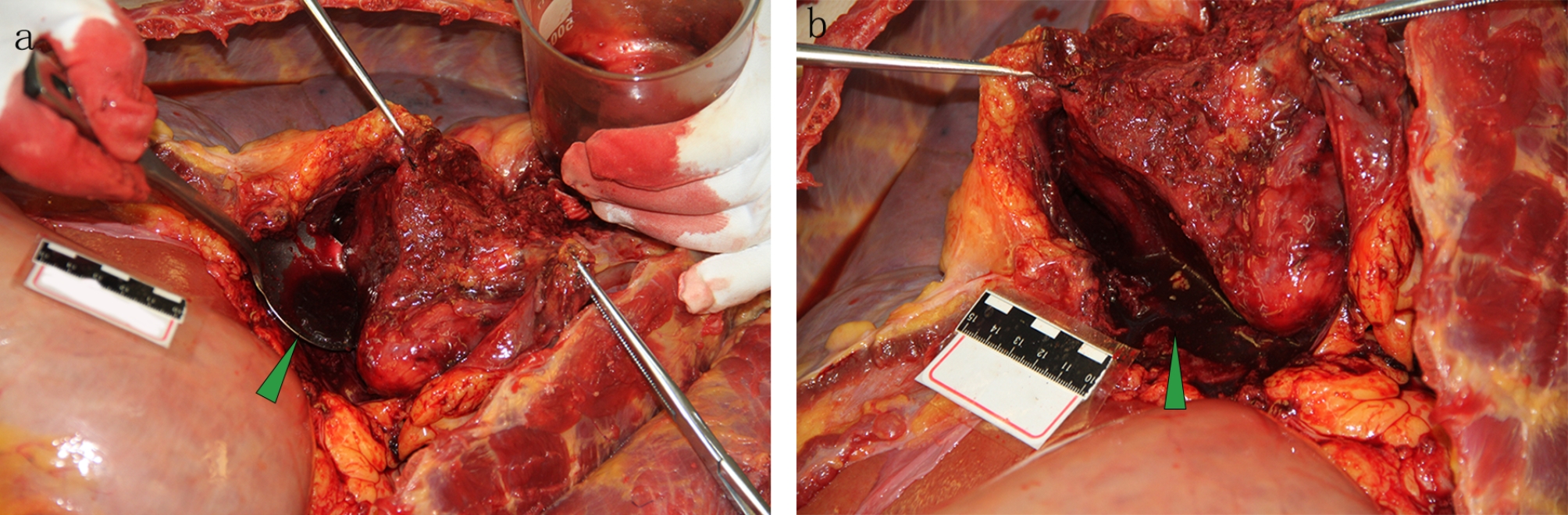
Autopsy findings. a and b. The pericardial and abdominal cavity was opened in a routine procedure. The results showed that a small amount of dark red fluid was accumulated in the pericardial cavity, which was thinner than blood

**Fig. 5:**
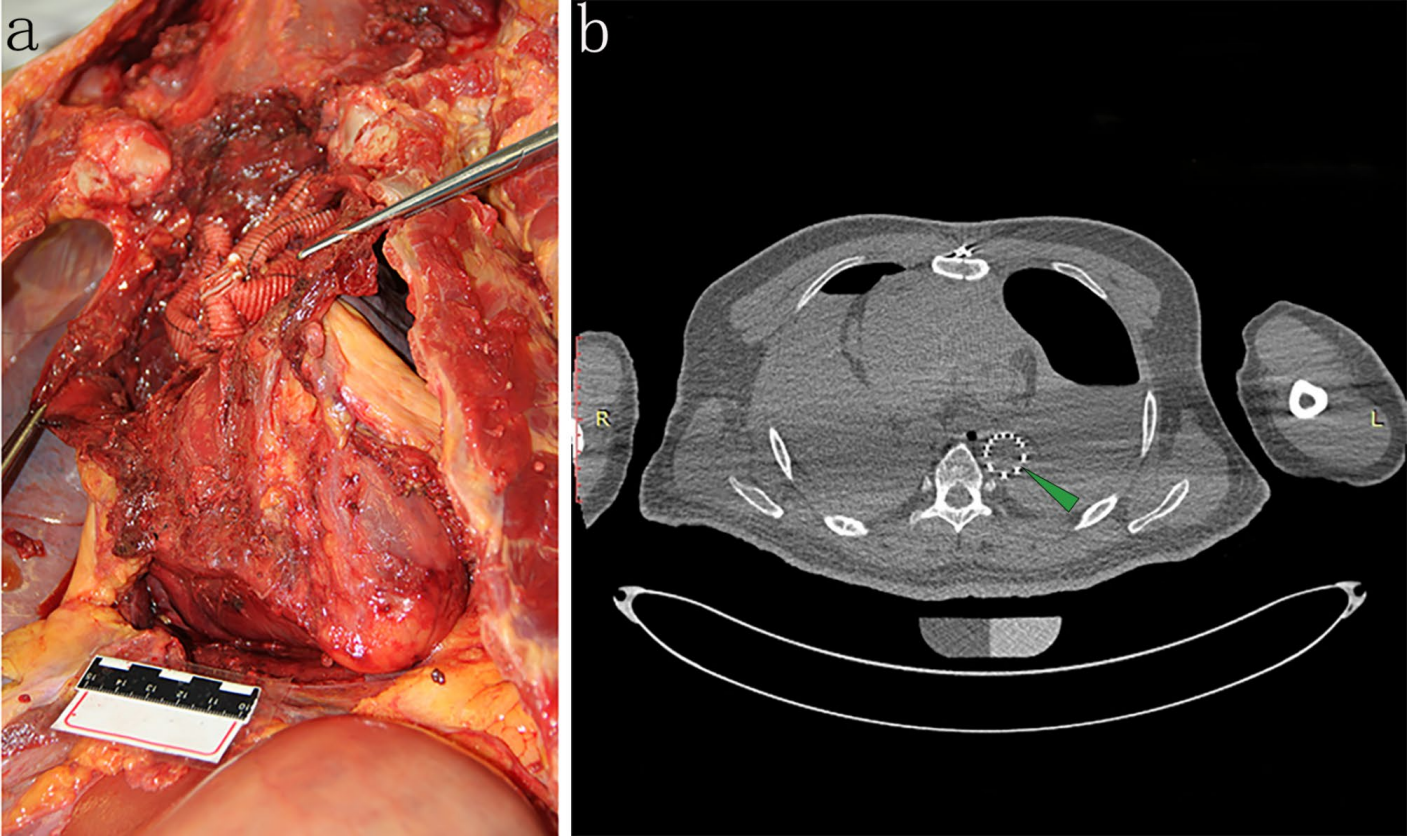
Autopsy findings. (**a**) The aortic arch was replaced by a stented elephant trunk artificial blood vessel and the incisions were tightly stitched. (**b**) The stented elephant trunk was in place

**Fig. 6:**
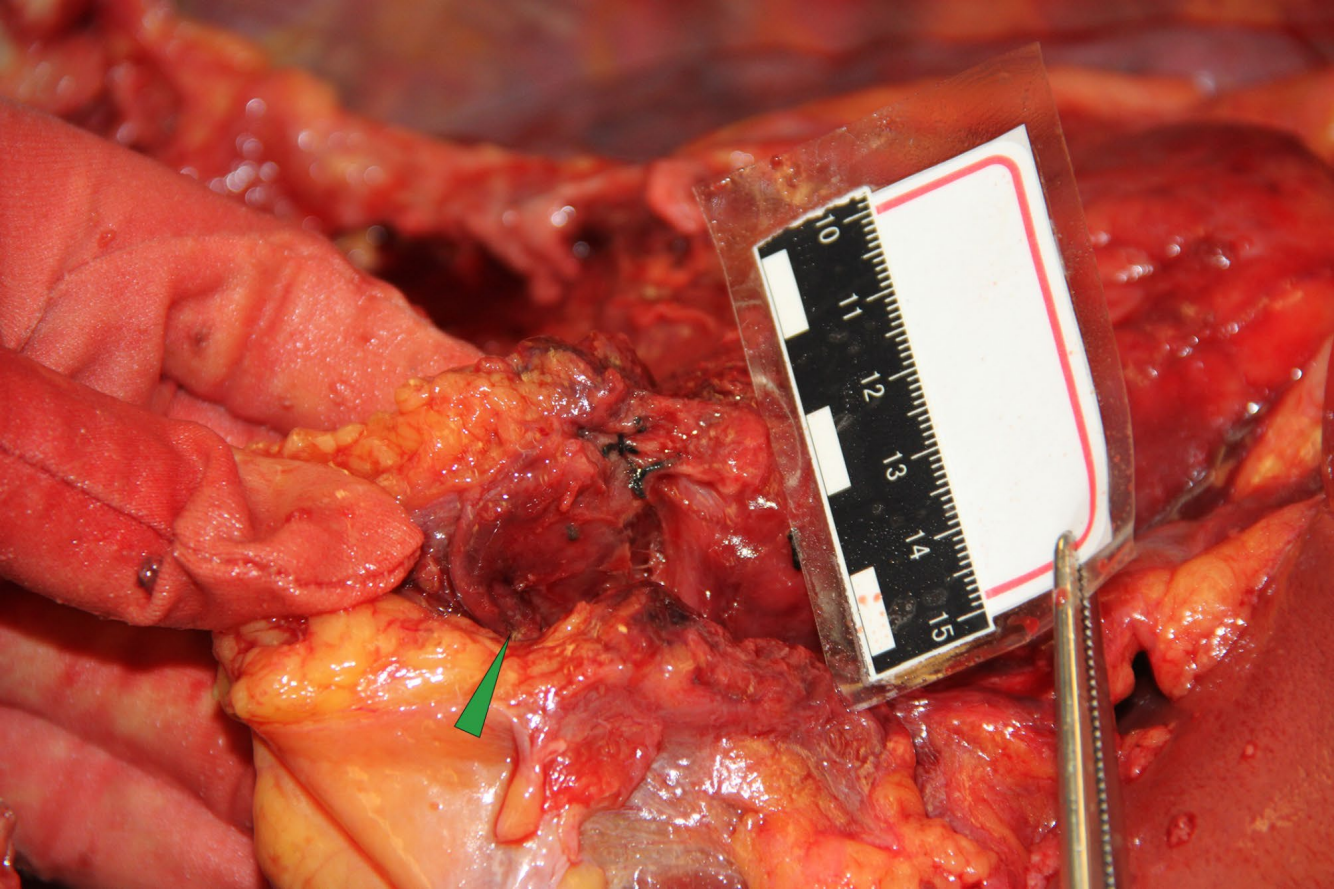
The pulmonary artery was also opened, and no thromboembolism was detected

The body was 166 cm tall with a medium build. There was a 22 cm incision on the middle chest that had already healed and two 1.5 cm sutured incisions, one on each side under the xiphoid process. The pericardial and abdominal cavity was opened in a routine procedure. Measurements showed that a small amount of dark red fluid accumulated in the pericardial cavity, which was thinner than blood (Fig. 4). The aortic arch was replaced by a stented elephant trunk artificial blood vessel, and incisions were tightly stitched. No obvious active hemorrhage was observed (Fig. 5). The pulmonary artery was also opened, but no thromboembolism was detected (Fig. 6).

**Fig. 7:**
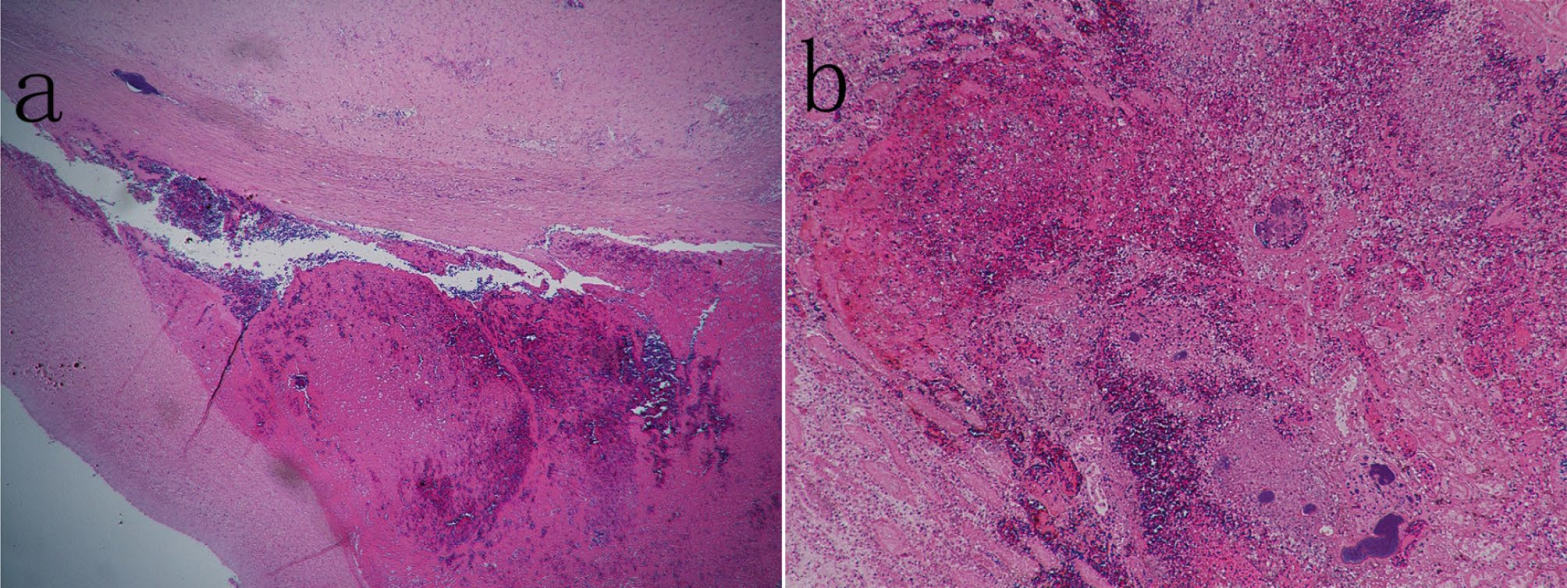
Histological findings. (**a**) Intimal tear with dissection formation near the anastomosis of the brachiocephalic trunk, and blood clots and thrombotic components were seen in the dissection. (**b**) Flaky necrosis with local small abscess of renal tissue

Histological findings (Fig. 7) show epicardial fibrous thickening, granulation tissue hyperplasia with inflammatory cell infiltration, and local necrosis. Part of the cardiomyocytes is hypertrophied, part of the myocardium is ruptured. Coronary atherosclerotic changes. thickening and fibrosis of the lining of the aorta, under which foam cells, cholesterol crystals and calcium salt deposits are seen; Intimal tear with dissection formation near the anastomosis of the brachiocephalic trunk, and blood clots and thrombotic components were seen in the dissection; Descending aortic dissection is formed, with a blood clot in the dissection at the beginning of the elephant trunk stent fixation, and less blood clots in the distal dissection at the beginning. Flaky necrosis of renal tissue with local small abscess were also observed.

### Cause of death

According to the CT scan, PMCTA, autopsy, and histological results combined with medical record, no bleeding was found that was caused by surgical sutures or postoperative incision’ s poor healing. Death from intracerebral hemorrhage, cardiac tamponade, or pulmonary embolism was also excluded. As he was diagnosed with heart, liver and kidney failure during his last hospitalization, consider histological results comprehensively, the cause of death was concluded to be multiple organ failure from postoperative heart, liver, and kidney insufficiency. The cause of such organ failure is related to the poor organ function of patients with heart disease and the need for long-term cardiac arrest and cardiopulmonary bypass maintenance during cardiac surgery, resulting in ischemia-reperfusion injury to the organ.

## Discussion and conclusions

Postoperative hemorrhage is one of the most common clinical complications [17, 18]. Because postoperative deaths often occur suddenly and unpredictably, they can lead to medical disputes between patients’ families and physicians. In such cases, the forensic investigation aims to determine if the death was caused by hemorrhage from incisions. This requires forensic pathologists to be familiar with the incision method and suture scheme of the surgery so during the autopsy they can detect if any malpractice in surgical suturing operation. Because of postoperative trauma repair and postmortem changes, it can be difficult to find significant result. Even if it is found, disputes often arise over whether the overflow occurred before death or if it was caused by instrumental pulling during autopsy.

Postmortem imaging technology is becoming increasingly integrated into forensic practices [12, 19]. For example, PMCT and PMCTA are frequently used to elucidate injury patterns and investigate cause of death [20–23]. PMCTA provided an effective solution for the case presented here. Because angiography was performed prior to autopsy, the malpractice in surgical incision could be ascertained by examining leakage of the contrast medium. If local leakage is present, then postoperative hemorrhage is suspected from surgical malpractice. Additionally, it can guide the forensic pathologist to focus on the suspected area during autopsy and further verify the positive imaging results. On the contrary, if there is no malpractice in incision, then there is no reason to believe that the surgical suture was controversial, thus removing considerations of medical malpractice.

5) This can cause controversy: if there exist malpractice caused by surgical sutures. For example, the malpractice of the surgical suture spacing. In a certain situation, the hemorrhage will only arise when the blood pressure reaches a certain level. If the blood pressure is reduced, then the incision is elastically retracted and closed and there will be no hemorrhage. This means that the injection of colored ink or a low-pressure contrast agent into the blood vessel is likely to result in the surgical sutures malpractice going undetected. For this case, the peristaltic pump was pressurized perfusion, simulating in vivo blood pressure levels. Therefore, the postmortem angiography evidence is more complete and convincing.

In conclusion, this case report provides a valuable approach for investigating medical disputes involving sudden death after cardiovascular surgery: (1) Performance of PMCTA before autopsy and (2) Simulating living state blood pressure using a peristaltic pump.

## Data Availability

Not applicable.
